# Non-Interfering Effects of Active Post-Encoding Tasks on Episodic Memory Consolidation in Humans

**DOI:** 10.3389/fnbeh.2017.00054

**Published:** 2017-03-29

**Authors:** Samarth Varma, Atsuko Takashima, Sander Krewinkel, Maaike van Kooten, Lily Fu, W. Pieter Medendorp, Roy P. C. Kessels, Sander M. Daselaar

**Affiliations:** ^1^Donders Institute for Brain, Cognition and Behaviour, Radboud UniversityNijmegen, Netherlands; ^2^Department of Neurobiology of Language, Max Planck Institute for PsycholinguisticsNijmegen, Netherlands

**Keywords:** episodic memory, consolidation, retroactive interference, resource allocation, reactivation, n-back tasks

## Abstract

So far, studies that investigated interference effects of post-learning processes on episodic memory consolidation in humans have used tasks involving only complex and meaningful information. Such tasks require reallocation of general or encoding-specific resources away from consolidation-relevant activities. The possibility that interference can be elicited using a task that heavily taxes our limited brain resources, but has low semantic and hippocampal related long-term memory processing demands, has never been tested. We address this question by investigating whether consolidation could persist in parallel with an active, encoding-irrelevant, minimally semantic task, regardless of its high resource demands for cognitive processing. We distinguish the impact of such a task on consolidation based on whether it engages resources that are: (1) general/executive, or (2) specific/overlapping with the encoding modality. Our experiments compared subsequent memory performance across two post-encoding consolidation periods: quiet wakeful rest and a cognitively demanding n-Back task. Across six different experiments (total *N* = 176), we carefully manipulated the design of the n-Back task to target general or specific resources engaged in the ongoing consolidation process. In contrast to previous studies that employed interference tasks involving conceptual stimuli and complex processing demands, we did not find any differences between n-Back and rest conditions on memory performance at delayed test, using both recall and recognition tests. Our results indicate that: (1) quiet, wakeful rest is not a necessary prerequisite for episodic memory consolidation; and (2) post-encoding cognitive engagement does not interfere with memory consolidation when task-performance has minimal semantic and hippocampally-based episodic memory processing demands. We discuss our findings with reference to resource and reactivation-led interference theories.

## Introduction

There is general consensus that wakeful rest after learning aids retention of episodic memories in humans—being able to remember our own personal past. During the post-learning rest period, recently acquired memories spontaneously reactivate by a replay of neuronal ensembles, triggered by hippocampal activity induced during the learning experience (Axmacher et al., 2008; Ego-Stengel and Wilson, 2009; Carr et al., 2011). Such reactivation of synaptic connections and circuits involved in the original memory formation gradually reinforce memory traces over time, leading to the persistent and gradual consolidation of episodic memories. This is reflected in the “Distributed Learning” effect: episodic learning sessions broken into separate segments—with wakeful rest breaks in-between—are more efficient as compared to a mass learning session (Cepeda et al., 2006; Ebbinghaus, 2013). From an educational perspective, these findings have been crucial in developing optimal learning strategies for students (Bloom and Shuell, 1981). Additionally, studies in the consolidation research domain also find that quiet wakeful rest periods after learning promote better memory retention, but presenting intervening cognitive tasks containing meaningful content causes forgetting by “retroactive interference” (Lechner et al., 1999; Dewar et al., 2009).

An open question, however, concerns the specific prerequisites for interference to occur. First, encoding contextually overlapping memory representations can cause interference. For example, in cue-overload paradigms (A-B, A-C learning), forgetting occurs simply by competitive replacement of one encoded item (B) by another item (C) associated to the same target (A) (Watkins and Watkins, 1975). A second type of interference arises from information that is contextually dissimilar to prior learning, such as in the case of everyday forgetting (Talamini et al., 2008). The primary cause of this is reallocation of brain resources from consolidation-relevant processes to activities involving environment monitoring, encoding and retrieval of information, language, emotion and sensorimotor processing, as well as social cognition. By resources, we refer to the overall energy budget of the brain, which is constant and limited (Raichle and Gusnard, 2002). It implies that attending to one task could drain brain resources away from performing another. With that in mind, a distinction can be made between tasks that reallocate brain resources from consolidation-related processes to cognitively demanding processes, and those that interfere with consolidation process through the use of resources common with the encoded material.

Given that the brain’s metabolic consumption remains stable across different brain states (Raichle and Gusnard, 2002), overloading the brain with resource-demanding tasks should cause task-irrelevant processes such as consolidation to be put on hold or suspended. Accordingly, post-encoding interference tasks such as, psychometric tests, “picture-search”, “spot-the-difference” etc., that rely on complex cognitive processing have been shown to cause significant levels of forgetting as compared to periods of quiet wakeful rest (Dewar et al., 2012; Craig et al., 2014). On the other hand, tasks involving secondary list learning and intentional autobiographical recall/future planning also cause interference (Craig et al., 2014), as they share specific resources with the encoded material owing to a common modality or long-term memory processing needs. Despite their dissimilarity to the original encoding, such tasks impinge not just on general resources, but also specific sensory-modal areas that, together with the hippocampus, hold the representations of the encoded material. Sustained competition for such specific resources could degrade existing representations and/or suppress reactivation of the encoded memory traces.

For interference to occur on memory consolidation, the purported task should therefore: (a) exhaust general resources for cognitive processing; or (b) inhibit ongoing reactivation in specific brain areas that are otherwise engaged in consolidation-relevant activities. By virtue of this fact, there have been no reports of post-encoding tasks that do not show an interference effect when compared to post-encoding rest. Interestingly, interference studies have only used tasks comprising of complex and meaningful stimuli that, as a function of their processing demands, trigger interference. Accordingly, the possibility of eliciting interference using a task that does not involve such multiple or overlapping processing has never been tested. Would engaging in a demanding task during the post-learning period interfere with memory consolidation if it: (a) requires only general resources for cognitive processing, or (b) requires resources shared with encoding but remains hippocampus-independent?

In order to address these issues, we employed a classical working memory task that has minimal semantic and hippocampal processing needs but high resource demands: the n-Back task (Owen et al., 2005). This task involves continuous monitoring and updating of presented stimuli. For each presented stimulus, participants have to indicate whether it matches the one from “n” steps earlier in the sequence. The load factor “n” can be adjusted to make the task more or less difficult. Our version of n-Back was set at a moderate level of difficulty (*n* = 2), involved numbers (1–5), and differed from traditional versions, in that it provided brief trial-by-trial feedback to prompt greater engagement and reduce task-unrelated thinking. In general, our paradigm involved post-encoding periods filled with quiet wakeful rest or n-Back task in a counterbalanced order, similar to the studies reported by Dewar and colleagues (Dewar et al., 2012; Craig et al., 2014). Subsequently, memory performance was tested using both free recall and associative-recognition tests. In a series of experiments reported here, we manipulated both the difficulty and the modality of the n-Back task to capture general and specific resources that might be engaged in the consolidation of recently encoded memories.

With the entirety of research into post-encoding states showing that mere engagement in a task can cause interference and only sleep-like states benefit consolidation, we hypothesized that much like any other non-rest task, n-Back too would certainly interfere with consolidation. It should draw executive resources away from proper maintenance or reactivation of memory traces. However, studies have also shown that 1-Back and 2-Back tasks are accompanied by suppression of hippocampal activity as compared to a non-memory guided sensorimotor baseline task such as 0-Back (Callicott et al., 2000; Esposito et al., 2006). Moreover, n-Back task can be performed just as well by patients suffering from episodic memory impairments (Snaphaan et al., 2009) and even hippocampectomy (Owen et al., 1996). Having no complex processing needs such as autobiographical recall, future scene construction or visual search etc., it is remarkably different from existing interference tasks. Being resource intensive, n-Back task might even promote an environment of reduced sensory and autobiographical stimulation during the consolidation period. As such, there is a small albeit real possibility that it might not interfere with consolidation. The existence of such a non-interfering “interference task” would challenge the prevalent notion that quiet rest is critical for memory consolidation in the awake state. This is an important issue for widening of our understanding of the relationship between rest, reactivation and cognitive resources involved in consolidation. From a distributed learning point of view, such a finding could give rise to a variety of tasks and learning techniques that would allow us to reduce environmental interference during post-study periods.

## Experiment 1

In Experiment 1, we investigated whether occupying general resources during post-learning period would hamper memory consolidation processes. The procedure comprised of two blocks of incidental encoding of word-picture pairs, each followed by a 12-min long consolidation period involving either wakeful rest (unfilled delay) or an n-Back task (filled delay) in a counterbalanced order, and ending with a delayed recognition memory test. We compared the effect of the rest period and the n-Back task on the consolidation of items learned prior to these delays. We hypothesized a saturation of general brain resources due to high and constant allocation of attention during the n-Back task, leading to a greater effect of interference as compared to the rest condition.

### Material and Methods

#### Participants

Forty-eight native Dutch, healthy students (32 female; *M*_age_ = 22.48, *SD* = 3.43), were recruited from Radboud University. Ten participants were removed from the study due to technical issues, non-conformance of protocol and low performance (d-prime) on the n-Back task (2-SD below the group average). Remaining 38 subjects were included in the analyses. After receiving written and oral instructions from the experimenter, all participants gave written informed consent in accordance with the Declaration of Helsinki. At the end of the experiment, participants received course credits or monetary compensation. This study was reviewed and approved by the Ethics Committee of the Faculty of Social Sciences of Radboud University.

#### Encoding Lists

Stimulus material consisted of 180 words and 180 pictures. All words were adjectives generated using the MRC Psycholinguistic database and subsequently translated into Dutch. Pictures of common objects, scenes or animals were downloaded from various image databases on the Internet. For each subject, the words and pictures were randomly paired and split into two lists for the two encoding blocks, each consisting of 90 unique word-picture pairs. Accordingly, the distribution of words and pictures was completely random across the two conditions, and for each subject.

#### Recognition Lists

The recognition list comprised of two types of trials—“identical/old” trials or “recombined/new” trials. In the “identical/old” trials, previously seen word-picture associations remained unchanged and were therefore identical to their encoded pairing. However, in the “recombined/new” trials, new pairs were presented by recombining old pairs. Within each encoding list, we shuffled half of the pairs and preserved the remaining 45 “identical/old” trials. This led to a total of 90 old pairs and 90 new pairs, which were randomly mixed together for the recognition memory test. Such recombination of pairs within each encoding list allowed us to calculate distinct memory scores (d-prime: Stanislaw and Todorov, 1999).

#### Procedure

**Figure 1:**
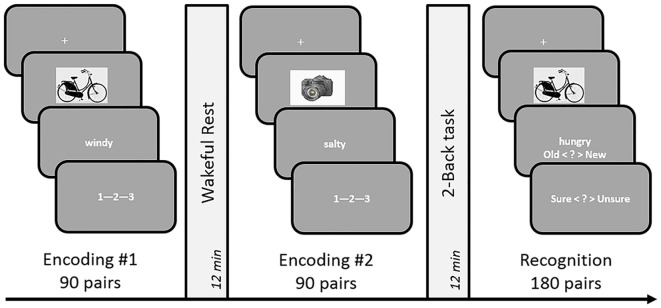
**Schematic design of Experiments 1 and 3.** The incidental encoding task involved associative decision making on object-word pairs, followed by a consolidation period occupied by either rest or a 2-Back task. The duration of these conditions was set to 12 min in Experiment 1 and 9 min in Experiment 3. A dynamic difficulty-adjusted version of the 2-Back task (DDA 2-Back) was used in Experiment 3. Subsequent to the two encoding-delay sessions, a surprise test of recognition memory was administered by presenting 180 object-words pairs that were either identical to the encoding sessions or recombined. The order of the rest and n-Back delay periods was counterbalanced across subjects.

In order to avoid usage of learning strategies and rehearsal during the encoding phase and post-encoding rest period, we employed an incidental encoding design for our experiments. Participants were recruited under the pretext of an experiment investigating emotional decision-making. The experimental design (see Figure 1) comprised of two associative decision making (encoding) tasks that were followed by 12 min of rest (unfilled delay) or 2-Back (filled delay) led consolidation periods, in a within-subject counterbalanced paradigm. Subjects were given detailed instructions and a short practice to acquaint them with the button presses and task requirements. The experiment was designed using PsychoPy presentation software (Peirce, 2009).

##### Encoding task

Each trial of the two incidental encoding blocks comprised of a decision-making task in which participants were required to associate each presented picture (e.g., “helicopter”) with its paired word (e.g., “thankful”). Participants were free to adopt any strategies like creating a vivid fictional story relating the two (e.g., “a family thanks the hospital’s air ambulance”) or by simply applying the word to qualify the picture. Subsequently, the presentation of a Likert scale “1—2—3” cued the subject to press an arrow key (“left”, “down” or “right”) on the keyboard to rate the vividness of their imagery judgment. Each trial began with the presentation of a fixation cross (0.5 s), followed first by a picture (1 s) and then its paired word (3 s). Trials were self-paced with maximum allowed response duration of 5 s.

##### Filled delay: 2-back task

One of the encoding sessions was followed by a delay of 12 min filled with the n-Back (*n* = 2) task with numbers (1–5). Each 2-Back trial started with a random grayscale number appearing in the middle of a dark screen for a maximum of 3 s during which the subjects were to press “right” if they had seen the number two trials earlier, or “left” otherwise. However, unlike traditional n-Back tasks, the grayscale number turned green (correct) or red (incorrect) for 300 ms showing a short feedback. The purpose of the feedback was to urge participants to keep their attention focused and induce optimal performance. No score or fixation-cross was displayed on the screen to avoid any visual interference. Participants were given detailed instructions and a short practice at the beginning of the experiment to acquaint them with the button presses and demands of the 2-Back task.

##### Unfilled delay: rest

During this post-encoding condition, subjects rested in the room for 12 min during which a fixation-cross remained on the screen. They were free to close their eyes, stare at the screen or look around the room and let their minds wander in a quiet wakeful state. The lights in the room were dimmed and the experimenter left the room to “prepare the next part of the study”.

##### Recognition task

At the end of the two encoding-delay periods, the experimenter informed the participant about the surprise memory test. The recognition task consisted of two blocks separated by an optional break. Each block ran through a randomized list of 45 “old/identical” and 45 “new/recombined” word-picture pairs, all of which contained the same words and pictures that were presented during encoding. The presentation of the recognition trials was identical to the encoding trials except that subjects now performed an associative recognition task in which they decided whether each presented pair was “old/identical” as seen during encoding, or “new/recombined”. On identifying an item as “old/identical”, participants were asked to indicate if they were confident of their response or not (“yes”/ “no”).

##### Analyses

In order to ensure that we included only those subjects who executed the 2-Back task at a reliable level, we excluded all participants who performed 2-SD below the group average on the d-prime score of this task. From the associative recognition task, hit rates of each list were calculated separately by dividing the number of correctly identified “old/identical” items by the total number of items to which the subject responded. False alarm rate of each list was similarly calculated by dividing the number of “new/recombined” items incorrectly identified as “old/identical”, by the total number of “new/recombined” responses. Only confident responses were included in this process to get an estimate of primarily recollection-based memory, avoiding trials performed with guesses. The standardized difference between hit and false alarm rates resulted in d-prime scores, representing subsequent memory performance (Stanislaw and Todorov, 1999). For statistical evaluation, we ran a paired-sample *t*-test with d-prime scores of rest and n-Back delay periods as the dependent variables. All results were analyzed using IBM SPSS 23, at an alpha level of 0.05.

### Results

**Figure 2:**
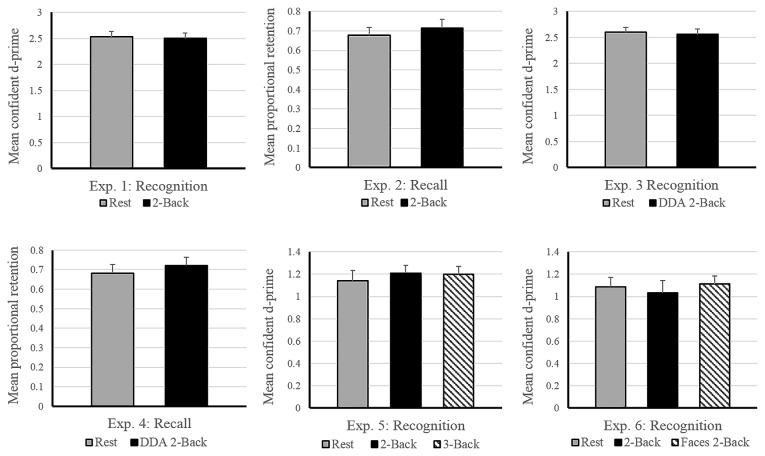
**Effects of post-encoding tasks on subsequent memory performance in Experiments 1–6.** The horizontal axis depicts the experimental design. Vertical axis shows memory performance measure involved in the corresponding experiment (d-prime: Experiments 1, 3, 5 and 6; Proportional Retention: Experiments 2 and 4). Experiments 3 and 4 involved dynamic difficulty-adjusted 2-Back task (DDA 2-Back). No effect of interference due to the n-Back task was found in either experiment. Error bars indicate standard error. Note: the scale of the graphs vary with differences in experimental design (2 vs. 3 encoding conditions) and performance measure (recall vs. recognition).

Average performance on the 2-Back task reached 91% accuracy, with a d-prime of 2.33 and reaction time of 0.93 s across subjects, suggesting that the participants were properly engaged in the n-Back task during the filled delay period (see Table 1). As for the memory performance of the word-picture association, Figure 2 shows that the d-prime memory scores of unfilled (rest: *M* = 2.52, *SD* = 0.64) and filled delay conditions (2-Back: *M* = 2.49, *SD* = 0.62), did not differ significantly; *t*_(37)_ = 0.33, *p* = 0.74.

**Table 1:** **Performance measures of the n-Back tasks**.

	Experiment 1	Experiment 2	Experiment 3	Experiment 4	Experiment 5		Experiment 6	
	2-Back	2-Back	DDA 2-Back	DDA 2-Back	2-Back	3-Back	2-Back	Faces 2-Back
d-prime	2.36 ± 0.47	2.14 ± 0.66	1.42 ± 0.37	1.45 ± 0.46	2.46 ± 0.85	1.34 ± 0.57	2.81 ± 0.64	2.79 ± 0.74
Accuracy	0.91 ± 0.03	0.88 ± 0.06	0.83 ± 0.05	0.83 ± 0.06	0.90 ± 0.05	0.82 ± 0.06	0.93 ± 0.03	0.93 ± 0.03
RT(s)	0.94 ± 0.23	0.79 ± 0.16	0.58 ± 0.18	0.68 ± 0.21	0.66 ± 0.18	0.71 ± 0.20	0.68 ± 0.20	0.69 ± 0.12

*n-Back task d-prime, overall Accuracy and average RTs across Experiments 1–6. Numbers represent mean and standard deviation*.

### Discussion

Results show that the two post-encoding delay periods did not differ in terms of subsequent memory performance: memory traces encoded prior to the 2-Back task underwent the same degree of consolidation as those encoded prior to rest. However, since we used a recognition memory test, this experiment may not have been sensitive enough to capture the differences in the strength of memory trace after a period of consolidation. Therefore, in order to ensure that our findings are reliable, we decided to test if they can be replicated in a strict free-recall paradigm.

## Experiment 2

The basic procedure of Experiment 2 was the same as that of Experiment 1 apart from the following. We changed the encoding materials from visually presented word-picture pairs to aurally presented words, the duration of the post-encoding delays was reduced from 12 min (in Experiment 1) to 9 min and the memory test applied was changed from recognition of word-picture pairs to free-recall of words. These changes allowed the study to be more similar to the studies conducted by Dewar et al. (2010) and Craig et al. (2014), who compared the effect of post-encoding rest period with various complex interference tasks.

### Material and Methods

#### Participants

Twenty native Dutch, healthy students (19 female, *M*_age_ = 21, *SD* = 2.19) were recruited from the Radboud University student pool. Two participants were removed from the analyses due to low n-Back performance using the same exclusion criterion as before (2-SD below mean group d-prime).

#### Encoding Lists

Instead of using visually presented adjectives as in Experiment 1, 40 commonly used nouns were recorded in the voice of a male native speaker of Dutch language. These words were chosen to have minimal semantic relatedness but matched on frequency and concreteness. For every participant, these 40 words were randomly split into two encoding lists of 20 words each.

#### Procedure

**Figure 3:**
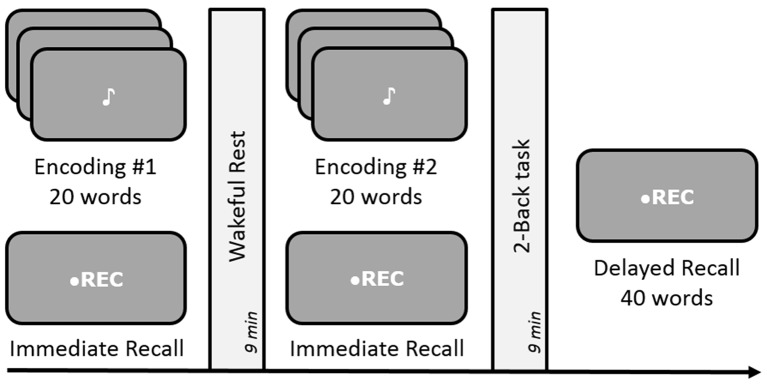
**Schematic design of Experiments 2 and 4.** Both experiments were conducted in a free-recall paradigm. Encoding involved memorizing and recalling a list of 20 words followed by either rest or a 2-Back task during the 9-min delay period for consolidation. Experiment 4 involved dynamic difficult-adjusted 2-Back task (DDA 2-Back). Subsequent memory of all 40 words was tested via an unexpected delayed recall test after the end of the two encoding-delay sessions. The order of the rest and n-Back delay periods was counterbalanced across subjects.

The experimental design was identical to standard experiments conducted by Dewar et al. (2010) and Craig et al. (2014). Each list comprised of 20 words, aurally presented every 2 s. Participants were instructed to carefully listen to and memorize each word for a quick test of their memory capacity, occurring immediately after the presentation of each wordlist. An immediate recall test was necessary to obtain a measure of initial memory retention as well as to avoid expectation of any future memory tests. After a 9-min delay, the second wordlist was presented followed by another immediate recall test corresponding to that list (see Figure 3). Subsequent to the second 9-min delay, an unexpected delayed free-recall test was administered to measure retention of the two lists across the two delay periods. As in Experiment 1, the two 9-min long delay periods were occupied by either a quiet wakeful rest (unfilled delay) or a 2-Back task (filled delay), in a counterbalanced order across participants. During both immediate and delayed recall tests, participants could recall as many words as possible, in any order. Both immediate and delayed free-recall responses were recorded on a mobile device and were scored offline.

### Analyses

For each wordlist, a proportional retention score was calculated by dividing the number of words recalled during delayed recall by those recalled during immediate recall. In case of perfect retention, the score was capped at 1. The immediate recall scores and the proportional retention scores of the two lists were used for the analyses. First, we ran a paired samples *t*-test on the immediate recall scores to verify that baseline memory performance did not differ across the two encoding sessions. Finally, in order to compare the effect of delay conditions on memory performance, we ran another paired samples *t*-test with proportional retention scores (rest vs. 2-Back) as dependent variables.

### Results

Immediate recall scores (rest: *M* = 11.05, *SD* = 3.29; 2-Back: *M* = 10.55, *SD* = 2.59) did not differ significantly between the two encoding blocks, (*t*_(17)_ = 0.919, *p* = 0.37), indicating that the quality of memory encoding did not differ across the two encoding sessions. As depicted in Figure 2, the mean proportional retention scores (rest: *M* = 0.67, *SD* = 0.17; 2-Back: *M* = 0.71, *SD* = 0.19) also did not differ significantly across the two delay conditions; *t*_(17)_ = −0.787, *p* = 0.44. Average performance on the 2-Back task across subjects reached 88% accuracy, with a d-prime of 2.14 and reaction time of 0.79 s.

### Discussion

Similar to Experiment 1, we observed no interference effect due to the 2-Back task in the experiment (see Figure 2). Across these two experiments, our results indicate that a post-encoding period filled with a cognitively engaging, working memory task such as n-Back, can be as conducive for consolidation as quiet wakeful rest. Although quiet rest has been shown to be favorable for consolidation during a wakeful state, our experiments provide the first evidence that it is not necessary.

It is possible that the n-Back task used in these experiments facilitated an environment of reduced sensory and autobiographical stimulation, unlike previously used interference tasks. During the debriefing session, participants generally reported a high degree of focus and involvement in the n-Back task interleaved with short episodes of task-unrelated mindwandering. Perhaps due to the monotonicity of the task, it is possible that participants were not challenged enough to constantly strain their general resources to interfere with the offline consolidation process. If this was the case, then our manipulation might not have been strong enough. Therefore, in order to make the n-Back task more stimulating, engaging and possibly interfering to consolidation, we modified its design by incorporating a trial-by-trial customization of difficulty to match with performance changes. In the next two experiments, the difficulty (response duration) of the n-Back task was dynamically adjusted at each trial according to subject’s cumulative performance. Integrating such dynamic difficulty adjustment could heighten the contrast between the filled and unfilled post-encoding delay periods leading to an interference effect.

## Experiments 3 and 4

Experiments 3 and 4 were replications of Experiment 1 (recognition) and 2 (free-recall) respectively, but the standard n-Back task was replaced with a dynamic difficulty-adjusted version, referred to as the DDA n-Back. According to our hypothesis, general resources are limited to be distributed between performing a working memory task and consolidating a set of recently acquired memories. As such a highly engaging and challenging DDA n-Back task should cause a significant reduction in memory performance as compared to quiet wakeful rest.

### Material and Methods

#### Participants

For Experiment 3, 38 participants (30 female; *M*_age_ = 22.29 years, *SD* = 3.12) and for Experiment 4, 32 participants (21 female, *M*_age_ = 26.34, *SD* = 3.05) were recruited from Radboud University. Ten participants were excluded on account of low performance on the DDA n-Back task or technical failures, leaving 36 participants in Experiment 3 and 24 in Experiment 4 for analyses.

#### Procedure

Across the two experiments, the design of encoding tasks, rest period, recall and recognition tests, general procedures and analyses remained identical to Experiments 1 and 2 respectively (see Figures 1, 3). However, the duration of post-encoding delay periods was set to 9 min and the 2-Back task was modified to DDA 2-Back, as mentioned in detail below.

##### Filled delay: DDA 2-back

Unlike the fixed 3 s response duration (speed) available in standard 2-Back used in Experiments 1 and 2, the dynamic difficulty-adjusted version (DDA 2-Back) had a variable speed that changed with the participant’s cumulative success at each trial. The initial and slowest speed of the task was preset at 2 s per item. When the participant’s score hit 80%, the speed increased at a rate of 0.2 s with each successful trial (1.8, 1.6, 1.4 and so on) until it reached the maximum threshold of 0.8 s. At peak performance, the participant only had 0.8 s to report to a 2-Back trial. The increase in the participant’s skill was therefore matched by a gradual increase in task difficulty. On the other hand, if the subject’s performance fell below 60%, the speed of the task decreased at a rate of 0.1 s and kept reducing until either it returned to the initial preset of 2 s, or performance recovered back to 60%. No changes were made when the score fluctuated between 60% and 80% as this was deemed to be the “flow zone”, where task difficulty was balanced by the subject’s skill and confidence (Nakamura and Csikszentmihalyi, 2014). By employing such dynamic difficulty adjustment, the participant could remain in control of the task, yet be adequately stimulated at all times.

### Results

As expected, the average reaction time on the DDA n-Back task was significantly faster (Experiment 3: 0.58 s and Experiment 4: 0.68 s) than its standard counterpart used in Experiment 1 (0.93 s) and Experiment 2 (0.79 s; see Table 1). In case of Experiment 3 (recognition design) results of the paired *t*-test showed no significant difference in the d-prime memory scores of rest (*M* = 2.59, *SD* = 0.57) and DDA 2-Back (*M* = 2.55, *SD* = 0.65) delay conditions; *t*_(35)_ = 0.54, *p* = 0.59. Similarly, in case of Experiment 4 (recall design), mean proportional retention scores (rest: *M* = 0.68, *SD* = 0.22; DDA 2-Back: *M* = 0.72, *SD* = 0.20) also did not differ significantly across the two encoding blocks; *t*_(23)_ = −0.64, *p* = 0.53 (see Figure 2 for results and comparison). Immediate recall scores (rest: *M* = 11.62, *SD* = 3.13; DDA 2-Back: *M* = 11.16, *SD* = 2.77) did not differ between the two encoding blocks; *t*_(23)_ = 0.74, *p* = 0.47, indicating that the quality of memory encoding was similar.

### Discussion

As shown under Table 1, participants in both Experiments 3 and 4 showed a severe taxation of brain resources to meet increased demands of the DDA n-Back task, as compared to Experiments 1–2. Results indicate that even when the difficulty of the n-Back task was continually fine-tuned to match participants’ growing competence at the task during the filled delay period, there was no effect whatsoever on the ongoing consolidation of memory. Despite imposing a constant deployment of resources during the 9-min delay, the effect of the post-encoding n-Back task did not differ from rest in terms of memory performance. Regardless, in a final attempt to further step up the resource load during the filled delay period, we ran another follow-up study comparing post-encoding rest with 2-Back as well as 3-Back tasks.

## Experiment 5

A possible explanation for the lack of interference effects could be that the encoding material used in Experiments 1–4 (word-picture pairs and wordlists) was quite easy to remember. Perhaps, our participants (all university students) were able to retain encoded information successfully over the n-Back delay periods by making conscious or subliminal within-list or extra-list associations. The other possibility is that the load of the n-Back task itself was not demanding enough to exhaust general cognitive resources for interference to occur. Therefore, in the following experiment, we addressed these two issues by: (1) making encoding material more challenging to retain (faces); and (2) increasing the load of the n-Back task (*n* = 3). Subjects encoded a large number of faces across three encoding blocks followed by one of three delay periods: rest, 2-Back or 3-Back tasks. In line with our original hypothesis, we expected a significant reduction in subsequent memory of faces learned prior to both n-Back tasks as compared to those learned prior to rest.

### Material and Methods

#### Participants

Forty native Dutch, healthy students (30 female, *M*_age_ = 22.26, *SD* = 2.59) were recruited from Radboud University. Four participants were removed due to technical difficulties or low n-Back task performance.

#### Encoding and Recognition Lists

Unlike the previous experiments, participants now performed an incidental face-encoding task. From the Chicago Face Database (Ma et al., 2015), we downloaded 270 images having equal number of male and female faces, and with a neutral expression. For each subject, 180 unique faces were randomly pooled and split into three encoding lists of 60 trials each. These 180 old faces and remaining 90 new faces were used for the recognition task. Half of both old and new faces were male.

#### Procedure

**Figure 4:**
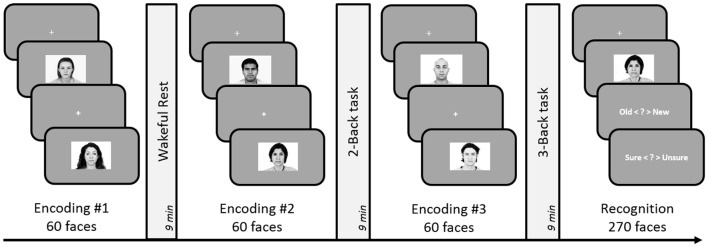
**Schematic design of Experiment 5.** Encoding session involved judgment of friendliness of presented faces. Post-learning delay periods were filled with either 2-Back, 3-Back or a rest period for 9 min. Subsequent to the three encoding-delay periods, a surprise recognition task was administered involving 180 old and 90 new faces. The order of the rest, 2-Back and 3-Back delay periods was counterbalanced across subjects.

The experiment consisted of three face-encoding tasks, each followed by a delay involving either rest, 2-Back or 3-Back task (see Figure 4). The three encoding-delay conditions were followed by a recognition memory task. The order of presentation of the three blocks was counterbalanced across participants. As before, participants underwent a short practice to familiarize themselves with button presses and task demands.

##### Face encoding task

For each encoding session, we asked the participant to judge the friendliness of 60 unique faces as they appeared on the screen for a fixed duration of 3 s. The participant rated the friendliness of the face using keys 1–4 (1 = “definitely unfriendly”, 4 = “definitely friendly”).

##### Delay periods: rest, 2-back and 3-back tasks

Similar to the previous experiments, one of the three post-encoding delay periods was filled with 9 min of quiet wakeful rest (unfilled delay). Two separate filled delay conditions were used in this Experiment: 2-Back and 3-Back tasks. The design of the n-Back tasks remained identical to Experiments 1 and 2 and ran for 9 min as well. DDA n-Back design was not used in this experiment as it could have caused a complete failure in carrying out the 3-Back task.

##### Face recognition task

For each of the 270 faces (180 old, 90 new) presented on the screen, participants indicated within 5 s, whether they had seen the face during the encoding session. Participants responded with keys Q and W (Q = “old”, W = “new”) on the keyboard. Subsequently, they had 3 s to indicate their confidence using keys 1–4 (1 = “definitely unsure”, 4 = “definitely sure”). As before, only confident responses (“definitely sure”) were considered for later analyses.

### Results

Calculation of n-Back performance and subsequent memory scores remained identical to Experiments 1 and 3. As expected, the average performance on the 3-Back task itself was significantly lower than on the 2-Back task (3-Back: d-prime = 1.34, Accuracy = 82%; 2-Back: d-prime = 2.46, Accuracy = 90%) but no difference in their overall reaction times was observed (see Table 1). In terms of memory performance of the three delay conditions, the d-prime memory scores (rest: *M* = 1.14, *SD* = 0.54; 2-Back: *M* = 1.21, *SD* = 0.45; 3-Back: *M* = 1.20, *SD* = 0.45) showed no significant difference on paired samples *t*-tests; rest vs. 2-Back: *t*_(35)_ = −0.91, *p* = 0.37, rest vs. 3-Back:* t*_(35)_ = −0.70, *p* = 0.49 and 2-Back vs. 3-Back: *t*_(35)_ = 0.15, *p* = 0.87 (see Figure 2).

### Discussion

Despite our attempts at exhausting brain resources across different designs of the n-Back task through Experiments 1–5, the post-encoding consolidation processes seem to linger on unaffectedly (see Figure 2). Although performance on the 3-Back task was significantly lower than on the 2-Back task (see Table 1 for a comparison), this did not cause any interference to consolidation of faces learned prior to the 3-Back task vs. the 2-Back task. Accordingly, one could argue that general brain resources may not be directly involved in wakeful consolidation, but perhaps in other, more peripheral processes (see Section “Resource Based Interference” under General Discussion). In the next experiment, we focused on exhausting specific resources, those involved during the original encoding episode.

## Experiment 6

As noted in the “Introduction” Section, post-encoding reactivation of memory traces is consequential to memory strengthening process. Forgetting could occur if reactivation is interfered by engaging encoding-specific resources in irrelevant tasks during the post-encoding consolidation period. In the following experiment, we tested this hypothesis by subjecting participants to a 2-Back task involving *faces*, after they encode a large number of face stimuli.

### Material and Methods

#### Participants

Twenty-seven native Dutch, healthy students (22 females, *M*_age_ = 22.52, *SD* = 2.27) were recruited from Radboud University. Three participants were removed from the analyses due to technical difficulties. Remaining 24 participants performed competently during the n-Back tasks involving numbers and faces, leaving no outliers.

#### Encoding and Retrieval Lists

Five new faces (3 female) downloaded from the Chicago face-database (Ma et al., 2015) were added to the stimulus set used in Experiment 5. We ensured that these pictures had a good mix of facial and hair features, similar to the faces seen during encoding.

#### Procedure

**Figure 5:**
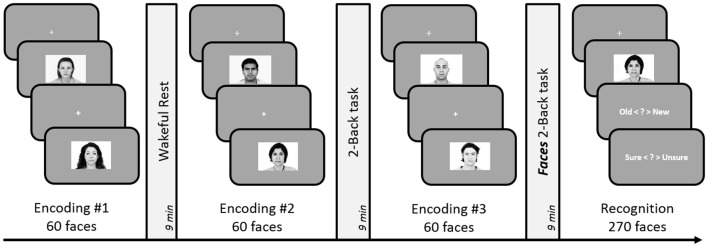
**Schematic design of Experiment 6.** Encoding session involved judgment of friendliness of presented faces. Post-learning delay period involved either 9-min of rest, 2-Back task (with numbers), or 2-Back task with faces. Subsequent to the three encoding-delay periods, a surprise recognition test involving 180 old and 90 new faces was administered. The order of the rest, 2-Back and Faces 3-Back delay periods was counterbalanced across subjects.

The experimental design and analyses remained identical to Experiment 5. However, we replaced the 3-Back task with a 2-Back task involving face stimuli (see Figure 5). The execution of the Faces 2-Back task was same as the 2-Back task used in previous experiments but involved five faces instead of five numbers: participants pressed one of two buttons indicating whether the currently displayed face was the same as the one they saw two trials ago.

### Results

Similar to previous experiments, the d-prime memory scores across the three delay conditions (rest: *M* = 1.08, *SD* = 0.39), 2-Back: *M* = 1.03, *SD* = 0.54, Face 2-Back: *M* = 1.11, *SD* = 0.38) showed no significant differences; rest vs. 2-Back: *t*_(23)_ = 0.63, *p* = 0.53, rest vs. faces 2-Back: *t*_(23)_ = −0.30, *p* = 0.76, and 2-Back vs. Faces 2-Back: *t*_(23)_ = −0.98, *p* = 0.33 (see Figure 2). Performance on the Face 2-Back task itself (*M* = 2.81, *SD* = 0.64) was also not significantly different from the 2-Back with numbers (*M* = 2.79, *SD* = 0.73) as revealed by a paired *t*-test: *t*_(23)_ = 0.20, *p* = 0.84 (see Table 1).

## General Discussion

This study compared active and passive post-learning periods with the purpose of investigating the specific prerequisites for interfering with the consolidation of episodic memories. Previous research has shown that post-learning engagement in any learning-unrelated task can cause forgetting, but periods of quiet wakeful rest facilitate retention of encoded stimuli. In these studies, the effect of a post-encoding period filled with quiet wakeful rest was compared with encoding-irrelevant tasks such as, psychometric tests, “picture-search”, “spot-the-difference”, secondary list learning, or autobiographical recall/future planning etc. (Dewar et al., 2012, 2014; Craig et al., 2014). The complex processing necessary for these tasks makes it difficult to determine which aspects actually interfere with post-learning consolidation. Accordingly, we exploited a less semantic task that is independent of the MTL memory system necessary for post-learning consolidation, but relies on our executive system instead: the n-Back task. More specifically, we addressed the question of whether successful memory consolidation could persist when memory encoding is followed by the n-Back task, which, on the one hand, is independent of hippocampal memory function and has minimal semantic content, but on the other hand, draws heavily on executive resources, as compared to a quiet wakeful rest period.

To address this question, we performed six experiments (total *N* = 176) manipulating different aspects of the n-Back task surrounding two critical issues in relation to memory consolidation: resources and reactivation. Our findings indicate that across different versions of the post-encoding n-Back task, subsequent memory performance did not differ from an equivalent period of post-encoding rest (see Figure 2), thereby suggesting that the items learned prior to these two distinct brain states achieved the same degree of memory consolidation. Null-findings, as demonstrated by the absence of an interference effect, can be challenging to address. Nonetheless, we find them reliable due to the replication of our results across six experiments involving different memoranda (word-picture pairs, wordlists and faces), task designs (difficulty adjusted n-Back tasks) and memory tests (free-recall and recognition). Although results reported from the recognition-based experiments (Experiments 1, 3, 5 and 6) were based only on confident d-prime, our findings remain unchanged when non-confident trials were also included in the analysis, thereby suggesting that there was no difference in memory accuracy across the two conditions. In contrast to existing literature, we found no empirical support for the notion that rest after learning is better for the fate of memory consolidation than any learning-unrelated cognitive engagement. In fact, when encoding is followed by a task that reduces sensory and autobiographical stimulation due to its high resource demands; and does not engage the hippocampal memory system, interference to memory consolidation does not occur. We interpret results of this study in the following sections with reference to resource-based and reactivation-based interference theories.

### Resource Based Interference

Given that the energy consumption of the brain remains relatively constant regardless of brain state and task engagement, brain resources need to be reallocated from one process to another depending on priorities of the task at hand (Raichle and Gusnard, 2002). Accordingly, one would expect that as compared to a quiet wakeful rest period, engaging in a task such as the n-Back during the post-learning period should take resources away from consolidation-relevant processes. In order to test this prediction, we conducted two experiments involving two different types of memory tests (Recognition: Experiment 1 and Recall: Experiment 2), both employing a standard 2-Back task with accuracy feedback. Even though the n-Back task places heavy demands on brain resources, our results show that this did not comply with the “resource-based interference” hypothesis. The task did not show any noticeable effect on memory consolidation: memory performance of items learned prior to the n-Back task remained the same as those learned before an equivalent period of rest.

Measurements of n-Back accuracy (see Table 1) indicated that our sample of participants (university students) were performing at a near ceiling level, and were perhaps not challenged constantly or sufficiently during the entire duration of the task. This led us to modify the design of the n-Back task on two different parameters: (1) Adjusting the difficulty of the n-Back task (DDA n-Back, Experiments 3 and 4) at each trial based on performance improvements, expecting that the brain will remain constantly occupied; and (2) Adding additional load (3-Back, Experiment 5) in an effort to exhaust participants’ resources as compared to the standard 2-Back task. Following the resource-based interference hypothesis, we predicted that such constant utilization of resources, or higher resources demands should lead to an interference effect relative to when participants can rest during the post-encoding delay period. Contrary to this assumption, neither of these manipulations elicited any effects of episodic memory interference.

At the outset, it appears as if consolidation is not a resource intensive process and can be suspended following irrelevant task demands. An alternative theory could be that wakeful rest itself may not be entirely optimal for consolidation. Andreasen et al. (1995) redefined REST as: “Rapid Episodic Silent Thinking”, a period of high cortical activity, including episodic memory processing (Buckner et al., 2008). Far from being a passive state, rest is accompanied with numerous, uncontrolled, highly active processes such as mentalizing, environment monitoring, mindwandering, autobiographical past and future thinking that involve a symmetrical network of the brain referred to as the Default-Mode Network (DMN: Gusnard and Raichle, 2001; Buckner et al., 2008). There is abundant evidence that such processes *accompany* consolidation during the wakeful resting state. In particular, mind-wandering in humans has been referred to as a subjective experience of memory reactivation, similar to dreaming during sleep (Christoff et al., 2011). However, there is no direct evidence that these processes actually *assist* consolidation: co-occurrence does not imply causality. In fact, post-encoding autobiographical thinking triggered by external cues has recently been shown to interfere with consolidation (Craig et al., 2014). These studies suggest that the demands of DMN-led ancillary processes during post-encoding rest could lead to a degree of interference that is comparable to that caused by the resource demands of the n-Back task. Our findings from Experiments 1–5 could therefore be explained from the perspective of this “*interference-account*” of resting-state processes.

An alternative account could be that regardless or perhaps due to its exhaustive resource load, the n-Back task indirectly *assists* memory consolidation by suppressing interference from internally generated cognitive activity. In addition to being independent of long-term memory-related hippocampal processing, demanding tasks including the n-Back have been shown to suppress the activity of the DMN (McKiernan et al., 2003; Daselaar et al., 2004; Esposito et al., 2006). As such, the n-Back task might, in theory, act as a cognitive barrier against interference from DMN-led processes that dominate resting state. Following from these two accounts, it is possible that—while n-Back suppresses DMN activity and prevents detrimental effects of ancillary resting state processes—the resultant memory facilitation gets canceled out by its pervasive resource demands. This might cause the effective degree of interference to be similar across post-encoding blocks of wakeful rest and demanding n-Back task, as evident from our first five experiments. In other words, it can be argued that the brain maintains similar degrees of consolidation across rest and n-Back states by balancing interference effects of the former by the resource demands of the latter.

### Reactivation Based Interference

Another critical issue regarding rest and consolidation is the concept of reactivation. Numerous studies demonstrated that neural activity patterns during encoding that are reactivated during periods sleep (McNaughton, 1998; Carr et al., 2011; Diekelmann et al., 2011) have also been observed during rest (e.g., Axmacher et al., 2008; Carr et al., 2011). According to the “Reactivation Based Consolidation” theory, such repeated reactivation of neural activity is critical to memory consolidation (McNaughton, 1998; McGaugh, 2000). In the post-encoding phase, activity pertaining to the encoded material “lingers on”. For example, Tambini et al. (2010) have provided evidence that post-encoding functional connectivity between the hippocampus and face-processing areas is correlated with subsequent memory of faces-object pairs. In Experiment 6, we targeted such encoding-specific areas by having participants process stimuli of the same category during the post-encoding consolidation phase. Instead of focusing on the resource-based interference effects studied in Experiments 1–5, this experiment was aimed at inhibiting specific resources (face processing areas, in our case) from reactivating previously learned material. In other words, we expected that consolidation should be hampered if there was an overlap between brain structures involved during the post-encoding task and consolidation-related reactivation process.

To this end, the n-Back task was modified to involve five faces (instead of numbers) and administered immediately after an incidental encoding task that also involved faces. Contrary to our expectation, but in line with the results of Experiments 1–5, the subsequent memory scores reflect that the Face n-Back task did not show any reduction in memory performance relative to when post-encoding period was occupied by rest. Several theories could account for the lack of interference effect in Experiment 6. From the perspective of the encoding modality, it can be argued that repeated presentations of the same five faces during the n-Back task did not engage the face-processing areas for a sustained period of time due to the “repetition suppression effect” (e.g., Goh et al., 2004; Summerfield et al., 2008). As a result, it is likely that the continuous processing of the n-Back task had only a temporary engagement with the face-processing areas, thereby sparing the reactivation of previously encoded faces. From the perspective of the hippocampus, two other possibilities emerge.

First, several studies have provided evidence that the hippocampus is not involved in the processing of the n-Back task (Callicott et al., 2000; Owen et al., 2005; Esposito et al., 2006), making it possible for recently encoded memory representations to remain preserved and their consolidation to go on uninterrupted. This evidence alone supports our findings. However, at least some degree of incidental encoding should have ensued during the n-Back task that allowed subjects to remember the episodic nature of the n-Back task including the memory of the stimulus, performance changes and their emotional state. All such processes could be potential sources of interference, similar to autobiographical thinking during rest. Given our results, the second possibility is that the degree of interference caused by incidental encoding during the n-Back task did not rise to the level of causing interference. Previously used tasks such as “picture-naming” or “autobiographical thinking” (Craig et al., 2014), which did show an effect of interference as compared to rest, involve much higher degrees of incidental encoding and sustained activation of the hippocampus and MTL in general (Buckner and Vincent, 2007; Christoff et al., 2009). Considering this evidence, our results suggest that the extent of hippocampal involvement during the n-Back task is comparable to or lower than that during the rest condition, even if the related memory representational areas were occupied during the post-encoding period. Thus, it seems that for interference to occur, a task or behavioral activity needs to have a large learning or retrieval component that persistently involves the hippocampus.

### Limitations and Future Directions

Due to the within-subject design employed in these experiments, it is understandable that there is a large difference between study-test intervals of the items encoded at the beginning vs. the end. For example, the consolidation of items in List 1 will be affected not just by the delay period immediately following the presentation of the list, but also by subsequent encoding of List 2, and the delay block afterwards. Accordingly, the effect of one delay period on the consolidation of a list cannot be completely isolated from that of the other. As such, this design may be less sensitive than a between-groups design in detecting differences between our conditions. A within-subject design can however be more sensitive in instances where the difference between conditions is relatively smaller than between-subject variations. As stated earlier, our experiments are derived from studies reported by Dewar et al. (2012) and Craig et al. (2014) who also used within-subject designs to show superior memory consolidation following periods of quiet wakeful rest than other irrelevant cognitive tasks. Similar to their studies, we also counterbalanced the delay type such that, both wakeful rest and the n-Back task conditions occur at different delay periods an equal number of times across subjects.

Another caveat of these experiments pertains to the duration of the consolidation period. Human and rodent studies that investigate the neurological basis of consolidation and the role of sleep, normally conduct memory tests atleast 12–48 h post-encoding, during which consolidation is said to have set in. Here, the scope of our investigation was limited to 9–12 min post-encoding, similar to other interference studies discussed earlier. Since we did not test memory the next day or a week later, we cannot claim that any long-term memory consolidation had occurred. While the time course of consolidation is a matter of ongoing research, several studies have successfully been able to tap into early consolidation processes using behavioral (Dewar et al., 2009; Tambini and Davachi, 2013; Craig et al., 2014; Sami et al., 2014), physiological (Cahill et al., 2003) and pharmacological (Cahill and Alkire, 2003) manipulations only minutes after a memory representation is encoded. Similarly, by inducing different brain states in the immediate post-encoding period, we attempted to capture any changes that occurred during the initial stages of memory consolidation. When tested over a longer timescale, whether memory performance of the items encoded prior to n-Back gradually declines (as compared to the wakeful rest condition), is a matter of future research.

Finally, due to its weak correlations with established measures of working memory span, there is some controversy surrounding the use of n-Back tasks in general (Kane et al., 2007). In particular, n-Back performance taps into both familiarity and recognition-based processes, which are not exactly representative of working memory (as compared with say, serial recall). However, none of these findings pertains to issues related to *long-term memory* processing and consolidation that are central to this study. Accordingly, irrespective of the performance characteristics of the n-Back task, its utility in this study comes from its non-complex nature and non-reliance on long-term memory/hippocampal processing. It remains to be seen whether we can replicate our results using other tasks that exhibit similar properties as the n-Back.

## Conclusion

The current study provides evidence that memory consolidation is not hampered by cognitive interference from tasks that have minimal semantic involvement and do not rely on hippocampally-based episodic memory processing. Our results suggest that, contrary to popular belief, wakeful rest is not necessary for consolidation. In fact, undergoing demanding cognitive tasks such as n-Back in the post-learning period lead to the same memory performance. These findings raise questions about the necessary prerequisites of a brain state to interfere with consolidation.

Rest is a period of highly complex uncontrolled activity involving episodic memory processes that could interfere with consolidation. Engaging in a demanding cognitive task such as n-Back in the post-learning period could, on the one hand, suppress these interfering processes, but, on the other hand, deprive consolidation of critical brain resources. One possible explanation could be that due to this balance between processes that facilitate and inhibit consolidation, the subsequent memory performance of items learned before a wakeful rest period or an equivalent n-Back task remains the same. In other words, it is possible that the brain maintains the ability to continue the consolidation process across passive and active states by balancing interference effects of the former by the resource demands of the latter.

Even though general resource demands did not seem to have an effect on memory consolidation, we tested whether post-learning recruitment of specific resources overlapping with the ones used during the learning episode should interfere with reactivation and consolidation. We did not find any evidence for this either. As noted before, one possible explanation is that a post-encoding task should engage the hippocampus in order to elicit memory interference, which is not the case for the n-Back task. The current data does not allow us to draw any strong conclusions that could answer these questions. In order to resolve the issues pertaining to resource recruitment and reactivation inhibition, we plan to use physiological and neuroimaging tools such as pupillometry and fMRI in future studies.

However, our results from six different experiments, involving different sets of participants and regardless of the type of learning tasks or memory tests, clearly show that rest is not a necessary prerequisite condition for successful consolidation. From an educational point of view, this study qualifies the notion of distributed learning by showing that rest-filled breaks are not essential. In fact, classroom learning episodes could be interleaved by entertaining prevocational or skill-learning tasks such as physical education, driving, sketching, music, cooking etc., which in the same vein as the n-Back task have: (1) a minimal overlap with previously learned memories of capitals and calculus; and (2) do not burden the long-term memory system. In short, there is no denying that quiet wakeful rest helps the mind and body in numerous ways, but to deem it necessary for consolidation is inaccurate and impractical.

## Author Contributions

SV, SK and MK were involved in data collection. SV, LF, SMD contributed to the design of the experiments. SV and SMD wrote the article with input from AT, WPM and RPCK.

## Conflict of Interest Statement

The authors declare that the research was conducted in the absence of any commercial or financial relationships that could be construed as a potential conflict of interest.

## References

[B180] Andreasen N. C. O’Leary D. S. Cizadlo T. Arndt S. Rezai K. Watkins G. L. (1995). Remembering the past: two facets of episodic memory explored with positron emission tomography. Am. J. Psychiatry 152, 1576–1585. 10.1176/ajp.152.11.1576 7485619

[B1] Axmacher N. Elger C. E. Fell J. (2008). Ripples in the medial temporal lobe are relevant for human memory consolidation. Brain 131, 1806–1817. 10.1093/brain/awn103 18503077

[B2] Bloom K. C. Shuell T. J. (1981). Effects of massed and distributed practice on the learning and retention of second-language vocabulary. J. Educ. Res. 74, 245–248. 10.1080/00220671.1981.10885317

[B4] Buckner R. L. Andrews-Hanna J. R. Schacter D. L. (2008). The brain’s default network: anatomy, function, and relevance to disease. Ann. N Y Acad. Sci. 1124, 1–38. 10.1196/annals.1440.011 18400922

[B3] Buckner R. L. Vincent J. L. (2007). Unrest at rest: default activity and spontaneous network correlations. Neuroimage 37, 1091–1096; discussion 1097–1099. 10.1016/j.neuroimage.2007.01.010 17368915

[B5] Cahill L. Alkire M. T. (2003). Epinephrine enhancement of human memory consolidation: interaction with arousal at encoding. Neurobiol. Learn. Mem. 79, 194–198. 10.1016/s1074-7427(02)00036-9 12591227

[B6] Cahill L. Gorski L. Le K. (2003). Enhanced human memory consolidation with post-learning stress: interaction with the degree of arousal at encoding. Learn. Mem. 10, 270–274. 10.1101/lm.62403 12888545 PMC202317

[B7] Callicott J. H. Bertolino A. Mattay V. S. Langheim F. J. Duyn J. Coppola R.. (2000). Physiological dysfunction of the dorsolateral prefrontal cortex in schizophrenia revisited. Cereb. Cortex 10, 1078–1092. 10.1093/cercor/10.11.1078 11053229

[B8] Carr M. F. Jadhav S. P. Frank L. M. (2011). Hippocampal replay in the awake state: a potential substrate for memory consolidation and retrieval. Nat. Neurosci. 14, 147–153. 10.1038/nn.2732 21270783 PMC3215304

[B9] Cepeda N. J. Pashler H. Vul E. Wixted J. T. Rohrer D. (2006). Distributed practice in verbal recall tasks: a review and quantitative synthesis. Psychol. Bull. 132, 354–380. 10.1037/0033-2909.132.3.354 16719566

[B11] Christoff K. Gordon A. Smith R. (2011). “The role of spontaneous thought in human cognition,” in Neuroscience of Decision Making, eds Vartanian O. Mandel D. R. (New York, NY: Psychology Press), 259–284.

[B10] Christoff K. Gordon A. M. Smallwood J. Smith R. Schooler J. W. (2009). Experience sampling during fMRI reveals default network and executive system contributions to mind wandering. Proc. Natl. Acad. Sci. U S A 106, 8719–8724. 10.1073/pnas.0900234106 19433790 PMC2689035

[B12] Craig M. Della Sala S. Dewar M. (2014). Autobiographical thinking interferes with episodic memory consolidation. PLoS One 9:e93915. 10.1371/journal.pone.0093915 24736665 PMC3988030

[B13] Daselaar S. M. Prince S. E. Cabeza R. (2004). When less means more: deactivations during encoding that predict subsequent memory. Neuroimage 23, 921–927. 10.1016/j.neuroimage.2004.07.031 15528092

[B14] Dewar M. Alber J. Butler C. Cowan N. Della Sala S. (2012). Brief wakeful resting boosts new memories over the long term. Psychol. Sci. 23, 955–960. 10.1177/0956797612441220 22829465

[B15] Dewar M. Alber J. Cowan N. Della Sala S. (2014). Boosting long-term memory via wakeful rest: intentional rehearsal is not necessary, consolidation is sufficient. PLoS One 9:e109542. 10.1371/journal.pone.0109542 25333957 PMC4198139

[B16] Dewar M. Della Sala S. Beschin N. Cowan N. (2010). Profound retroactive interference in anterograde amnesia: what interferes? Neuropsychology 24, 357–367. 10.1037/a0018207 20438213 PMC2864945

[B17] Dewar M. Garcia Y. F. Cowan N. Della Sala S. (2009). Delaying interference enhances memory consolidation in amnesic patients. Neuropsychology 23, 627–634. 10.1037/a0015568 19702416 PMC2808210

[B18] Diekelmann S. Büchel C. Born J. Rasch B. (2011). Labile or stable: opposing consequences for memory when reactivated during waking and sleep. Nat. Neurosci. 14, 381–386. 10.1038/nn.2744 21258327

[B19] Ebbinghaus H. (2013). Memory: a contribution to experimental psychology. Ann. Neurosci. 20, 155–156. 10.5214/ans.0972.7531.200408 25206041 PMC4117135

[B20] Ego-Stengel V. Wilson M. A. (2009). Disruption of ripple-associated hippocampal activity during rest impairs spatial learning in the rat. Hippocampus 20, 1–10. 10.1002/hipo.20707 19816984 PMC2801761

[B21] Esposito F. Bertolino A. Scarabino T. Latorre V. Blasi G. Popolizio T.. (2006). Independent component model of the default-mode brain function: assessing the impact of active thinking. Brain Res. Bull. 70, 263–269. 10.1016/j.brainresbull.2006.06.012 17027761

[B22] Goh J. O. Siong S. C. Park D. Gutchess A. Hebrank A. Chee M. W. (2004). Cortical areas involved in object, background and object-background processing revealed with functional magnetic resonance adaptation. J. Neurosci. 24, 10223–10228. 10.1523/JNEUROSCI.3373-04.2004 15537894 PMC6730187

[B23] Gusnard D. A. Raichle M. E. (2001). Searching for a baseline: functional imaging and the resting human brain. Nat. Rev. Neurosci. 2, 685–694. 10.1038/35094500 11584306

[B24] Kane M. J. Conway A. R. Miura T. K. Colflesh G. J. (2007). Working memory, attention control, and the N-back task: a question of construct validity. J. Exp. Psychol. Learn. Mem. Cogn. 33, 615–622. 10.1037/0278-7393.33.3.615 17470009

[B25] Lechner H. A. Squire L. R. Byrne J. H. (1999). 100 years of consolidation—remembering Müller and Pilzecker. Learn. Mem. 6, 77–87. 10327233

[B26] Ma D. S. Correll J. Wittenbrink B. (2015). The chicago face database: a free stimulus set of faces and norming data. Behav. Res. Methods 47, 1122–1135. 10.3758/s13428-014-0532-5 25582810

[B27] McGaugh J. L. (2000). Memory—a century of consolidation. Science 287, 248–251. 10.1126/science.287.5451.248 10634773

[B28] McKiernan K. A. Kaufman J. N. Kucera-Thompson J. Binder J. R. (2003). A parametric manipulation of factors affecting task-induced deactivation in functional neuroimaging. J. Cogn. Neurosci. 15, 394–408. 10.1162/089892903321593117 12729491

[B29] McNaughton B. L. (1998). The neurophysiology of reminiscence. Neurobiol. Learn. Mem. 70, 252–267. 10.1006/nlme.1998.3851 9753600

[B30] Nakamura J. Csikszentmihalyi M. (2014). “The concept of flow” in Flow and the Foundations of Positive Psychology, ed. Csikszentmihalyi M. (Netherlands: Springer), 239–263.

[B31] Owen A. M. McMillan K. M. Laird A. R. Bullmore E. (2005). N-back working memory paradigm: a meta-analysis of normative functional neuroimaging studies. Hum. Brain Mapp. 25, 46–59. 10.1002/hbm.20131 15846822 PMC6871745

[B32] Owen A. M. Morris R. G. Sahakian B. J. Polkey C. E. Robbins T. W. (1996). Double dissociations of memory and executive functions in working memory tasks following frontal lobe excisions, temporal lobe excisions or amygdalo-hippocampectomy in man. Brain 119, 1597–1615. 10.1093/brain/119.5.1597 8931583

[B33] Peirce J. (2009). Generating stimuli for neuroscience using PsychoPy. Front. Neuroinform. 2:10. 10.3389/neuro.11.010.2008 19198666 PMC2636899

[B34] Raichle M. E. Gusnard D. A. (2002). Appraising the brain’s energy budget. Proc. Natl. Acad. Sci. U S A 99, 10237–10239. 10.1073/pnas.172399499 12149485 PMC124895

[B35] Sami S. Robertson E. M. Miall R. C. (2014). The time course of task-specific memory consolidation effects in resting state networks. J. Neurosci. 34, 3982–3992. 10.1523/JNEUROSCI.4341-13.2014 24623776 PMC3951697

[B36] Snaphaan L. Rijpkema M. van Uden I. Fernández G. de Leeuw F.-E. (2009). Reduced medial temporal lobe functionality in stroke patients: a functional magnetic resonance imaging study. Brain 132, 1882–1888. 10.1093/brain/awp133 19482967

[B37] Stanislaw H. Todorov N. (1999). Calculation of signal detection theory measures. Behav. Res. Methods Instrum. Comput. 31, 137–149. 10.3758/bf03207704 10495845

[B38] Summerfield C. Trittschuh E. H. Monti J. M. Mesulam M. M. Egner T. (2008). Neural repetition suppression reflects fulfilled perceptual expectations. Nat. Neurosci. 11, 1004–1006. 10.1038/nn.2163 19160497 PMC2747248

[B40] Tambini A. Davachi L. (2013). Persistence of hippocampal multivoxel patterns into postencoding rest is related to memory. Proc. Natl. Acad. Sci. U S A 110, 19591–19596. 10.1073/pnas.1308499110 24218550 PMC3845130

[B41] Tambini A. Ketz N. Davachi L. (2010). Enhanced brain correlations during rest are related to memory for recent experiences. Neuron 65, 280–290. 10.1016/j.neuron.2010.01.001 20152133 PMC3287976

[B39] Talamini L. M. Nieuwenhuis I. L. Takashima A. Jensen O. (2008). Sleep directly following learning benefits consolidation of spatial associative memory. Learn. Mem. 15, 233–237. 10.1101/lm.771608 18391183

[B42] Watkins O. C. Watkins M. J. (1975). Buildup of proactive inhibition as a cue-overload effect. J. Exp. Psychol. Hum. Lear. Mem. 1, 442–452. 10.1037//0278-7393.1.4.442

